# *CFTR* Mutation Analysis in Western Iran: Identification of Two Novel Mutations

**Published:** 2018

**Authors:** Nasibeh Karimi, Reza Alibakhshi, Shekoufeh Almasi

**Affiliations:** 1- Department of Animal Biology, Faculty of Natural Sciences, University of Tabriz, Tabriz, Iran; 2- Department of Biochemistry, School of Medicine, Kermanshah University of Medical Sciences, Kermanshah, Iran; 3- Department of Biology, Faculty of Life Science, Dalhousie University, Halifax, Nova Scotia, Canada

**Keywords:** *CFTR* gene, Cystic fibrosis, Iran, Middle East, R334W

## Abstract

**Background:**

Cystic fibrosis (CF) is one of the most common autosomal recessive disorders in Caucasian population. The incidence of disorder varies among different religious, ethnic and geographical isolates. The aim of this study was to identify the spectrum and the frequency of known and unknown disease-causing mutations in Iranian CF patients.

**Methods:**

Genomic DNA was extracted from peripheral whole blood with a QIAamp DNA Mini-Kit. Mutation analysis was done in the *CFTR* gene including complete coding region and intron/exon boundaries using a direct sequencing method.

**Results:**

In general, ten mutations were identified in 27 CF cases. Two out of 10 mutations, 754delT and GGTGGCdel/TTGins, were reported as novel mutations. The most common observed mutations in patients were R334W (40.74%), ΔF508 (18.5%), K710X (12.96%) and D110H (5.5%), 1897C>G (1.85%), R1162X (1.85%), S466X (1.85%) and T1036I (1.85%).

**Conclusion:**

The finding indicated a unique mutation panel which can be used in genetic counseling, prenatal diagnosis and future screening of CF in Iran. Although ΔF508 is the most common mutation in other populations including Caucasian, this mutation seem not to have an important role in Iranian CF patients. Findings suggest that a different approach in molecular genetics diagnostic strategies in Middle Eastern countries including Iran should be considered.

## Introduction

Cystic fibrosis (CF; MIM # 219700) is a life-threatening autosomal recessive disorder with prevalence of around 1 in 2500 births in Caucasian population. However, its incidence varied based on ethnic and geographical background. CF is caused by mutation in Cystic Fibrosis Transmembrane Conductance Regulator (*CFTR*; #602421) gene which encodes a small ATP- and cAMP-dependent chloride channel placed on the apical border of epithelial cells of intestine, respiratory systems, pancreas, gall bladder, and sweat glands (1). The *CFTR* gene is located on the chromosomal region 7q31.2 and contains 27 exons which cover about 250 *kb* (2). Malfunction of *CFTR* channel leads to manifestation of multisystem diseases including airway disease, pancreatic failure, meconium ileus, male infertility and elevated levels of Na and Cl in sweat (3, 4). Worldwide investigation showed an increase in the number of CF causing mutations to reach 1850. ΔF508 mutation is the most common mutation which has been observed in approximately two- thirds of CF patients. Although other mutations are rare, founder effect increases the frequency of certain mutations among a population (2, 5, 6). Therefore, knowledge about frequency and distribution of CF mutations in each population can be beneficial in disease management, development of diagnostic tools and prenatal diagnosis Due to difficulty of sequencing the entire *CFTR* gene, clinical laboratories need a test system which can screen a panel of the most prevalent mutations in each population. Therefore, it is crucial to determine the frequency and the spectrum of the *CFTR* gene mutations in different populations especially highly mixed ones. Molecular analysis of the *CFTR* gene in countries with prevailing Caucasian population is already well determined but there is minimal knowledge about CF prevalence in Asia and specifically in Iran. Historically, being a link between Africa, Europe, India and beyond makes Middle East, especially Iran, an area with genetically mixed population. Therefore, the present study was designed to explore the distribution of *CFTR* gene mutations and polymorphisms in the Kurdish population of the Kermanshah province using direct DNA sequencing. Detecting disease causing mutations and polymorphisms in this population may shed light on the most common disease causing mutations outside Africa. In addition, these findings could be used in parental diagnosis.

## Methods

### Patients:

CF patients from all regions of Kermanshah province were referred to the medical genetic laboratory of Kermanshah University of Medical Sciences, Kermanshah, Iran over a period of two years. Only patients with a classical form of CF including lung disease with or without pancreatic insufficiency who had two positive sweat tests (cut-off,60 *mmol/l*) were included in the current study. In total, 27 unrelated Kurdish families with an affected child were analyzed. All CF cases were native of Kermanshah province and they had been there for at least three generations.

### Analysis of mutation and polymorphism:

*CFTR* gene mutation study was conducted on 27 unrelated, ethnically Kurdish CF patients who were born in Kermanshah province. After taking 10 *ml* blood, DNA was extracted from peripheral blood samples by using salting out precipitation method.

Mutation detection analysis was performed in complete coding region and exon/intron junctions of patients by direct sequencing method. All extracted DNA samples were sequenced exon by exon. The main criteria of exon selection were the total number of present mutations and worldwide frequency of mutations in each exon.

First of all, *CFTR* gene’s fragments were amplified by PCR using a GeneAmp PCR System 9700 (Applied Biosystems, USA). PCR conditions were as follows: initial denaturation at 95°*C*, 5 *min*: each cycle; denaturation at 95°*C*, 45 *s*, annealing at 56–61°*C* (depending on primers’ Tm), 45 *s*, elongation at 72°*C*, 69 *s*, 30 cycles: final elongation at 72°*C*, 7 *min*. The agarose gel was stained with ethidium bromide for visualizing the fragment migration.

For sequencing analysis, samples were analyzed by direct sequencing of all 27 exons of the *CFTR* gene and their flanking introns in an ABI-3130 DNA analyzer (Applied Biosystems, USA). PCR products were purified using QIA quick PCR purification kit. Following this, samples were precipitated with Ethanol–Sodium Acetate precipitation and were used for cycle sequencing. Sequencing and data analysis were done by using ABI Prism 310 DNA sequencer (Applied Biosystems, New Jersey, USA) and sequencing analysis software version 5.2, respectively. For patients carrying the novel and R334W mutations, parental DNA samples were sequenced to confirm the results.

## Results

### Families:

A total of 27 unrelated Kurdish families were screened in this study (13 males and 14 females; aged between 2 months and 19 years). In total, 18 patients were included because of their high degree of consanguinity amongst Iranian populations. Around 80% of patients were suffering from malnutrition, and fatty stool while 81.4% patients were diagnosed with congenital meconium ileus. Moreover, all patients had either a severe or slight respiratory problem. The average sweat test value for the patients was 105 *mmol/L*.

### Mutation detection:

During mutation screening of 27 Iranian CF patients (54 chromoosomes), ten different *CFTR* mutations were detected; R334W was the most frequent one (40.74% of CF alleles). All ten mutations cover almost 90.7% of CF alleles in patient group. Two out of 10 mutations were 754delT in exon 13 and GGTGGCdel/TTGins in 14b exon; these novel results have not yet been reported anywhere around the world. The most prevalent mutation worldwide is ΔF508 and it was observed in 18.5% of patients. Other mutations were as follows: K710X (12.96%) (in press), D110H (5.5%), 754delT (1.85%), R1162X (1.85%), 1897C>A (1.85%), S466X (1.85%) and T1036I (1.85%). Four polymorphisms which were detected in patients during mutation screening were M470V (74.1%), 3897A>G (12.96%), 2694T>G (5.55%) and 4389 G>A (3.7%). These experiments were repeated several times for confirmation. DNA sequencing which was done on blood samples of R334W patients’ parents showed heterozygosity for R334W locus. The identified mutations of the patients in this study are shown in table 1.

**Table 1.** Frequencies of *CFTR* mutations identified in studied patients

**Gene location**	**Mutation**	**Legacy name**	**No. of patients**		**Total alleles**	**Global distribution**
			**Homo**	**Hetero**		
**Exon 7**	c.1132C>T	p.R334W	10	2	22(40.74%)	Southern European, Latin American
**Exon 13**	c.2128A>T	p.K710X	2	3	7(12.96%)	Southern French
**Exon 14b**	c.2619int	GGTGGCdel/TTGins	-	1	1 (1.85%)	Novel
**Exon 4**	c.460G>C	p.D110H	1	1	3(5.55%)	Southern European
**Exon 13**	c.2261delT	p. 754delT	-	1	1 (1.85%)	Novel
**Exon 19**	c.3616C_T	p.R1162X	-	1	1(1.85%)	Native American
**Exon 10**	c.1652del3	p. ΔF508	2	6	10 (18.5%)	Global
**Exon 10**	c.1529C>G	p.S466X	-	1	1 (1.85%)	Turkish, Greek
**Exon 17b**	c. 3239 C>T	p.T1036I	-	1	1 (1.85%)	Iran (rare)
**Exon 13**	c. 1897C>A	p. L633I	-	1	1 (1.85%)	rare

Novel mutations appear in bold. Variants are described using the DNA and protein designation: cDNA level (c.), amino acid changes at the protein level (p.)

### Novel mutations 754delT and GGTGGCdel/TTG ins:

The novel mutation 754delT was discovered in exon 13 of the *CFTR* gene. In this mutation, a thymine at the 2261 nucleotide was deleted which in consequence formed a stop codon. As a result, a truncated protein was formed thus abolishing the channels function. A 15-year-old male patient (with 754delT) was diagnosed with CF in the first 3 months of his life. He had positive sweat test of 150 *mmol/l* and suffered from respiratory problem, pancreatic sufficiently and meconium ileus.

**Figure 1.**
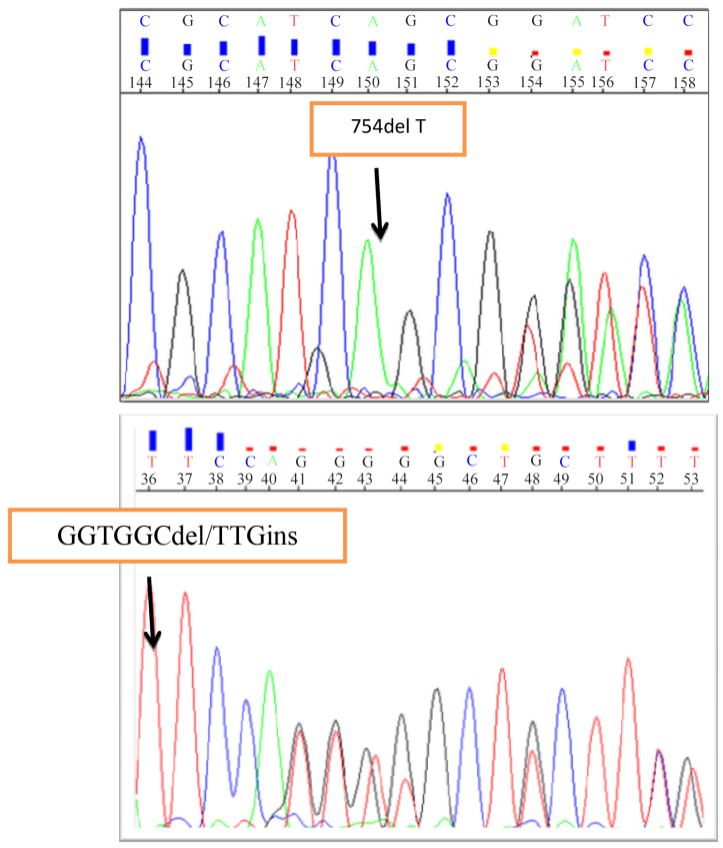
Novel mutations were identified in *CFTR* gene by sequencing

Another novel mutation, GGTGGCdel/TTGins, was identified in exon 14b. In this mutation, a sequence containing six nucleotides, GGTGGC, was deleted at the intron-exon junction. It means that 1 nucleotide in 5′-end of 13th intron and 5 nucleotides (GTGGC) in 3′-end of exon 14b (2619 to 2624) were deleted. Insertion of TTG at the start point of the exon 14b replaced valine and alanine amino acids with leucine (TTG) which results in a frame shift mutation and production of an abolished channel (Figure 1).

## Discussion

This study is the first to report the prevalence of *CFTR* mutations in CF patients from west part of Iran with Kurdish ethnic background. Unfortunately, data on live births were not registered in Iran, so computation of the prevalence of CF is impossible in Iran. Nevertheless, previous reports showed that CF isn’t rare in Asia, especially in Iran (7–12). Reports have shown that frequency and distribution of the *CFTR* gene mutations varies between Iranian population and its neighboring countries such as Pakistan, Turkey and Arabian countries (13–17). As CF mutations have high heterogeneity in Iranian population, sequencing and multi mutation screening aren’t fully useful, so there are few data regarding CF in Iran (11, 18). The important objective of diagnostic analysis is to provide a chance for families at risk to get a prenatal diagnosis. Thus, the purpose of the present study was diagnosis and prevention of CF occurrence through mutation screening of *CFTR* gene in CF patients with Kurdish ethnic background.

In this study, R334W mutation was the most frequent mutation between the tested patients (40.74%). Almost all patients who carried R334W in both homozygote and heterozygous forms had respiratory problems, pancreatic insufficiency and high sweat test with diagnosis age of below 1 year. R334W mutation is a quite prevalent CF causing mutation among CF patients of east Mediterranean countries including Crete, Spain, France, Cuba and Mediterranean France with occurrence rates equal to 11.5%, 5%, 0.42%, 5.2% and 1.63%, respectively (19–22). R334W occurrence was also reported in Brazil, Latin America, Poland, Greece, Romania, Germany, Czechs, and Ukraine (23–27). R334W mutation has been previously reported in other provinces of Iran with incidence of 2.9% and 0.89% (10, 11, 18). Frequency of R334W mutation in the present study (40.74%) was significantly greater than the worldwide frequency. It can suggest the greater prevalence of R334W mutation among Mediterranean population which is passed to west Asia particularly Middle East. Similarly, previous studies on beta thalassemia display a mixture of Asian and European alleles in Iran (28). Our findings were also consistent with the latest findings about PAH gene in Kermanshah province, although characteristics of PAH mutations were different between studied provinces and other parts of the Iran and Mediterranean region (29). As Morral et al. had shown the R334W mutation has different origins; it’s probable that it has different and independent origins in our studied population too.

ΔF508 is the most common CF mutation in the world and accounts for 70% of CF mutations (2). Interestingly, it was found that ΔF508 is the second most common mutation with 18.5% incidence in studied population (published data). Worldwide investigation showed that frequency of ΔF508 mutation reduces from north (87.2% in Denmark) to south (24–27% in Turkey) of Europe, so due to geographical situation, it is expected that ΔF508 frequency in Iran will be lower or equal to its frequency in Turkey. Based on our results, the frequency of ΔF508 mutation in Kurdish CF patients was lower than European countries (19, 30).

K710X was the third most common mutation with a frequency of 12.96 %. K710X mutation hasn’t been previously reported both in Iran and Asia (13, 16, 31, 32). K710X mutation has been detected in Mediterranean France (0.93%), Spanish ancestry (0.56%) and Finland (1%) (20, 21, 33, 34). Due to the special geographical location of Iran in relation to both Asia and European continents, being placed in west of Asia and south east of Europe, it has been suggested that Iranian population receives some of the prevalent mutations from both continents. Based on this idea, K710X mutation might have been introduced to Iran from European countries.

The forth prevalent mutation was D110H which was found in 5.55% of CF patients. This mutation has high frequency in Southern Europe, Turkey, Italy and Iran (6, 11, 14, 35, 36). It is possible that D110H mutation in Iranian population was originated from Turkey.

The rest of detected mutations had frequencies below 2%. Two of them had low worldwide frequencies and two remaining mutations were novel mutations. R1162X mutation had 1.85% frequency in our population. Although geographical distribution of R1162X mutation is mainly focused in Italy, among Amerindians and Latin Americans, it happens in low frequency in other regions too, such as Iran, Mediterranean France and Spain (18, 20, 21, 25, 28, 37). Previously, Alibakhshi et al. have shown the occurrence of R1162X mutation in Kermanshah province (18). Consequently, the mutation may be common in this province but its low frequency was due to its low sample size.

1897C>A mutation had 1.85% frequency in CF patients. For the first time, 1897C>A was discovered in a Portuguese patient who carried none of CF causing mutations in other exons (38). Similarly, in our study, a patient was only carrying 1897C>A mutation.

Interestingly, two novel CF causing mutations including 754delT and GGTGGCdel/TTGins were found with the same frequency of 1.85%.

The next identified mutation was S446X with 1.85% frequency. S446X mutation is a rare mutation in the world that was identified in Greece and Turkey in low frequency (6). Unlike other countries, the frequency of the former mutation in Iran was reported as 5.8%. As S446X mutation was previously reported in other provinces of Iran, it might be a common mutation in Iran. T1036I, the last identified mutation with 1.85% frequency in the studied population was also reported in Iran before. Finally, 3897 A>G polymorphism is a rare synonymous variant in the *CFTR* gene which was identified with a high frequency in our population (39). It is wonderful to note that all patients who were carrying K710X mutation had 3897 A>G polymorphism too.

In summary, 23 out of 27 patients had two mutations in their *CFTR* gene, two patients were identified with a single mutation and the other two had no *CFTR* mutation. The controversial result of two patients with no CF causing mutation could be due to the presence of mutation in the other region of gene or having less than 100% mutation efficiency. Additionally, those patients were suspected to have CF due to having a sweat test result close to the borderline.

Given the fact that high worldwide diversity in frequency and spectrum of the *CFTR* mutations was reported, our findings were likely a consequence of a combination of two causes: firstly, there is a trend among Kurdish families to marry within family and secondly, Kermanshah province’s geographic proximity to the Mediterranean region could be the other reason.

ΔF508 mutation is the most common mutation among almost all populations, but there are still other mutations with a higher frequency in the other parts of the world such as in the Middle East. It is worth mentioning that different and unique spectrums of mutations in *CFTR* gene were observed in non-European population such as in Kashmiri population of India. A recent study in Chinese CF patients also showed that G970D is the most common mutation with a high frequency in studied patients (40). In addition, a study in Oman reported p.S549R as the most frequent mutation in that population with a frequency of 65.2% (41).

It is well known that different ethnic groups have their own unique and different *CFTR* mutation panel that may include one or a few common founder alleles. R334W is much more prevalent in Kermanshah than in any other population in which it has been identified. The unexpectedly high prevalence of this mutation may be interpreted by consanguinity and other factors like genetic drift. Furthermore, the two *CFTR* mutations, R334W, K710X, may be specific to the culturally homogenous Kurdish population. On the other hand, significant differences were found between the distribution and frequency of some identified mutations between studied population and others. In addition, some mutations such as R1162X and S466X previously reported among Iranians, were identified in the present study.

Kurds are the most common ethnic group in Kermanshah and have existed in the area from the first millennium BCE. Due to the special geographical location of Iran in relation to both Asia and European continents, being placed in the route of major ancient movements of the Caucasian people towards the Mediterranean basin, it has been suggested that Iranian population received some of the prevalent mutations from both regions. Therefore, most of the identified mutations in studied patients are prevalent in the Mediterranean region; our findings are in agreement with the historical and geographical features of Kermanshah.

## Conclusion

Our study has been successfully extended to prenatal diagnosis and screening program in studied population and four mutations (R334W, ΔF508, K710X and D110H) with total proportion of 77.7% were suggested for molecular analysis among studied populations. According to our results, the Iranian population has a unique distribution of *CFTR* gene mutations, so sequencing the entire *CFTR* gene is suggested for mutation analysis in Iranian population.
